# Supplementary material for: Characterisation of Bee Pollen from the Marche Region (Italy) According to the Botanical and Geographical Origin with Analysis of Antioxidant Activity and Colour, Using a Chemometric Approach
Source: Molecules. 2022 Nov 18;27(22):7996. doi: 10.3390/molecules27227996 (PMC9692707; doi:10.3390/molecules27227996)
Supplement: Supplementary file 1 [file molecules-27-07996-s001.zip › molecules-2012510-supplementary.pdf]

Table 1S. Complete palynological analysis of the bee pollen samples. Identification of pollen type and percentage, of harvesting zone (Isola del Piano = PU; Loc. Cavaceppo = AP; Matelica = MC) and period for each pollen sample are provided.
The botanical species was determined also using the knowledge on territory’s flora, the cultivation maps, and beekeepers’ indications.

**Code**	PU-FR01	PU-FR02	PU-FR03	PU-FR04	PU-FR05	PU-CO06	PU-CO07	PU-CO08	PU-CO09	AP-SA01	AP-FR02	AP-FR03	AP-QU04	AP-VI05	AP-QU06	AP-QU07	AP-CA08	AP-CA09	AP-CA10	AP-CA11	AP-CA12	MC-TR01	MC-TR02	MC-TR03
**Period**	Apr 22	Apr 26	Apr 29	May 03	May 06	Jun 28	Jul 01	Jul 03	Jul 07	Apr 21	Apr 30	May 08	May 15	May 22	May 29	Jun 05	Jun 12	Jun 19	Jun 26	Jul 03	Jul 09	Jul 07	Jul 09	Jul 12
Acer	5										5													
Castanea																8	36	76	84	100	100			
Cercis	10	5																						
Cistus incanus																	10	7						
Cistus salvifolius													5											
Clematis																	7	6						
Compositae Forma S																						1	<1	<1
Cruciferae																						<1	<1	5
Erica										8														
Fraxinus ornus	57	51	64	73	63					13	68	44												
Genista f.														5										
Graminaceae < 35 µm																								<1
Helianthemum f.																						2	2	1
Helianthus f.																						<1		<1
Hypericum t.																								<1
Lotus																							1	2
Olea f.																41	35							
Onobrychis																						11	16	13
Papaver			8	8									6											<1
Plantago																						<1	1	<1
Prunus f.	9									23		31												
Pyrus/Malus f.		5									10	14												
Quercus ilex gr.													66	36	76	41								
Quercus robur gr.		21	18	6						7														
Robinia					12																			
Rubus f.						10	7	10	5													23	16	21
Salix	7									40														
Trifolium alexandrinum						24	11	7														46	55	50
Trifolium repens gr.																						10	6	3
Umbelliferae Forma A						63	73	76	80													6	3	5
Vitis					10									50	11									
